# *Bifidobacterium animalis* subsp. *lactis* A6 alleviates perennial allergic rhinitis in adults by inhibiting serum total IgE and IL‐13: A randomized, double‐blind, placebo‐controlled trial

**DOI:** 10.1002/clt2.70064

**Published:** 2025-06-12

**Authors:** Langrun Wang, Shiwen Zhou, Huiyu Chen, Chao Zhang, Meiwen Sun, Qi Zhang, Yinghua Liu, Shaoqi Shi, Shaoyang Ge, Juan Chen, Yanling Hao, Yong Zhang, Bing Fang, Jingjing He, Ran Wang

**Affiliations:** ^1^ Department of Nutrition and Health China Agricultural University Beijing China; ^2^ Key Laboratory of Precision Nutrition and Food Quality Ministry of Education China Agricultural University Beijing China; ^3^ Department of Nutrition The First Medical Center of Chinese PLA General Hospital Beijing China

**Keywords:** *Bifidobacterium animalis *subsp. *lactis *A6, immunoglobulin E, interleukin‐13, perennial allergic rhinitis

## Abstract

**Objectives:**

The evidence regarding the efficacy of probiotics in improving allergic rhinitis (AR) remains inconsistent. This study aimed to evaluate the potential effects of *Bifidobacterium animalis* subsp. *lactis* A6 (A6) on perennial AR.

**Methods:**

A randomized, double‐blind, placebo‐controlled trial was conducted involving 70 adults with perennial AR receiving either probiotic (A6, 5 × 10^10^ CFU/sachet per day) or placebo intervention for 8 weeks. Nasal symptoms and quality of life (QoL) were recorded using total nasal symptom scores (TNSS) and the rhinitis quality of life questionnaire (RQLQ). Blood eosinophil count, total immunoglobulin E (IgE), allergen‐specific IgE, and immunological parameters were also assessed.

**Results:**

After 8 weeks of intervention, the probiotic group showed a statistically significant greater reduction in TNSS total score compared with the placebo group [−3.11 (3.53) vs. −1.29 (3.34), *p* = 0.029, Cohen's *d* = 0.68]. Similar results were noted for serum total IgE and interleukin‐13 (IL‐13). Comparable findings were seen for RQLQ score only at week 4 but not at week 8.

**Conclusions:**

In conclusion, A6 could statistically significantly alleviate rhinitis symptoms and improve QoL in adults with perennial AR. The effect size, as measured by Cohen's *d*, suggests that A6 may provide clinically meaningful benefits for AR patients to a certain degree.

**Clinical Trial Registration:**

Chictr.org.cn Identifier no. ChiCTR2200064158.

## 1 INTRODUCTION

Allergic rhinitis (AR) is one of the most common chronic diseases globally, often lasting a lifetime. Nasal congestion, rhinorrhea, nasal itching, and sneezing are characteristic symptoms of AR,^1^ resulting in impaired quality of life (QoL), numerous comorbidities,^2^ unproductivity at work^3^ and economic burden.^4^ The prevalence of AR has increased rapidly in both western and eastern adults,^5^, ^6^ affecting at least 500 million people worldwide, with conservative estimates.^1^

Immunoglobulin E (IgE) and T helper (Th)2 cell signature cytokines (e.g., interleukin‐4, interleukin‐5, interleukin‐9, and interleukin‐13^7^) are key mediators in AR pathology. After nasal mucosa exposure to allergens, plasma cells produce allergen‐specific IgE (sIgE) that sensitize target cells (e.g., eosinophils), and subsequently trigger AR. Simultaneously, Th2 cell signature cytokines can aggravate this process.^8^ A reciprocal inhibitory relationship exists between Th1 cell signature cytokines (e.g., interferon‐γ) and regulatory T (Treg) cell signature cytokines (e.g., transforming growth factor‐*β*) and Th2 cell signature cytokines.^7^ Additionally, IL‐10 is typically considered an inhibitor of Th2 cells in allergic reactions^9^, ^10^ despite being produced by various cell types.^11^

Pharmacotherapy and allergen‐specific immunotherapy are the main clinical treatments for AR^12^; however, they have some limitations (e.g., adverse effects).^8^ Growing clinical evidence has proven the effectiveness of probiotics in alleviating AR.^13^, ^14^, ^15^ The most recent meta‐analysis (2022) also demonstrated the benefits and safety of probiotics in patients with AR; however, it revealed high heterogeneity in results.^16^ Many observational studies have discovered that gut microbiota play an important role in AR onset.^17^, ^18^, ^19^ To our knowledge, only a few randomized controlled trials (RCTs) have systematically measured changes in the balance of Th1/Th2/Treg cytokines after probiotic intervention.^20^, ^21^ Thus, further investigations are required to examine probiotics' efficacy on rhinitis symptoms and QoL while considering their impacts on AR patients' immune balance.

The abundance of *Bifidobacterium* in the intestine has emerged as a promising target for allergic diseases, owing to its potential role in regulating host immune homeostasis.^22^ Interventions with specific strains of *Bifidobacterium* ^23^, ^24^ were associated with superior relief of AR symptoms and improved QoL compared with other probiotics, as indicated by meta‐analysis results.^16^ *Bifidobacterium animalis* subsp. *lactis* A6 (A6, CGMCC NO.9273) is a probiotic bacterium isolated from centenarians' feces. Research showed that A6 exhibits high tolerance to low pH, crucial for its health‐promoting properties.^25^ In vitro safety assessments of A6 revealed no safety concerns,^26^ and clinical trials have indicated its capacity to modulate the human gut microbiome.^27^ Recent animal studies have further explored the potential of A6 in regulating metabolism and inflammation.^28^ Studies also proved that A6 significantly elevates IL‐10 levels while reducing IL‐13 levels.^29^, ^30^ These findings highlight A6's potential in immune balance regulation and its possible role in alleviating AR symptoms. Therefore, it is meaningful to assess the efficacy of A6 on AR to fully determine its therapeutic potential. As a result, this study will conduct an RCT to evaluate A6's effects on rhinitis symptoms, QoL, and immune homeostasis in patients with AR.

## 2 MATERIALS AND METHODS

### 2.1 Study design

A randomized, double‐blind, placebo‐controlled trial was performed in Beijing, China to investigate the effects of A6 on AR. Anti‐AR medications were prohibited during the trial. This study was approved by the Institutional Review Board of the China Agricultural University Ethics Committee (CAUHR‐20220906). Each participant signed an informed consent form before trial commencement. The study was registered at Chictr.org.cn (registration number: ChiCTR2200064158).

### 2.2 Sample size calculation

Sample size calculation was based on the primary outcome measure, total nasal symptom scores (TNSS). According to Singh et al.,^23^ after an 8‐week intervention of *Bifidobacterium lactis* NCC2818 and placebo, the TNSS value was 1.50 ± 1.33 in the probiotic group and 3.00 ± 2.04 in the placebo group. In order to achieve a statistical power of 80% with a significance level (alpha) of 0.05, the sample size required for each group was 22 as calculated using PASS 15. In consideration of an approximately 20% drop‐out rate, 30 patients were planned to be recruited in each group.

### 2.3 Inclusion and exclusion criteria

Inclusion criteria^1^: individuals aged 18–65 years; and^2^ exhibiting ≥2 symptom domains of rhinitis (Nasal congestion, rhinorrhea, nasal itching and sneezing), with scores ≥2 in the absence of medication; and^3^ having a history of AR lasting for more than 1 year; and^4^ demonstrating at least one positive result for the four most common perennial inhalation allergens (*Dermatophagoides pteronyssinus* (*Dp*), *Dermatophagoides farine* (*Df*), *cat*, and *dog*) in Beijing^31^ (sIgE ≥0.35 kU/L, using ImmunoCAP 250 (Phadia)).

Exclusion criteria^1^: individuals diagnosed with allergic diseases other than AR (e.g., dermatitis, asthma); or^2^ those with concurrent upper respiratory tract infections, sinusitis, or nasal polyps; or^3^ pregnancy or lactation; or^4^ with a history of other serious disorders (e.g., congenital immune diseases, cardiovascular, hepatic, renal, cerebral, hematopoietic system disorders, mental illness, or tumors); or^5^ with milk allergies or lactose intolerance; or^6^ use of antihistamines or nasal corticosteroids within 1 week prior to the start of the study, or use of oral corticosteroids in the 3 months prior to the study; or^7^ have ever received immunotherapy (e.g., allergen‐specific immunotherapy); or^8^ using any anti‐AR medication.

### 2.4 Randomization and blinding

From September 2022 to October 2022, 70 patients with perennial AR were enrolled in this study (Implementation personnel: L.W., Q.Z., S.Z., C.Z., M.S., Y.L., S.S., S.G., J.H., and R.W.). A statistical analyst (C.Z.) conducted sample random allocation by generating a computer‐based random sequence. Participants were randomly assigned (1:1) to the probiotic group and the placebo group (Implementation personnel: J.H. and L.W.). All study personnel and participants were blinded to the group assignments during the whole intervention. The intervention substances in the two groups were identical in appearance, smell, and taste.

### 2.5 Intervention and procedures

**FIGURE 1:**
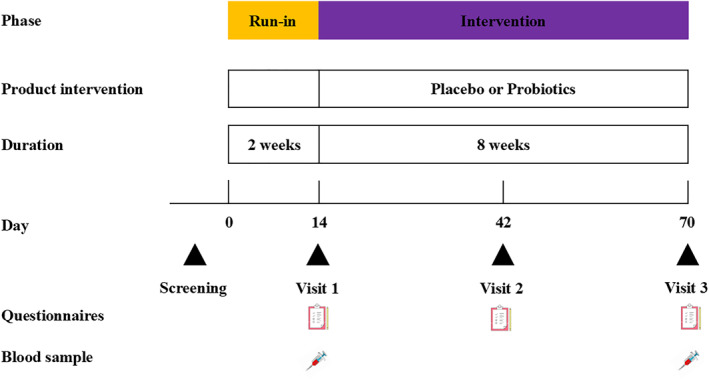
Study design. Probiotic: *Bifidobacterium animalis* subsp. *lactis* A6. Placebo: maltodextrin. Visit1: Baseline, Visit 2: Week 4, Visit 3: Week 8.

This study was conducted between 22nd October 2022 to 31st December 2022, including a 2‐week run‐in period and 8‐week intervention period (Figure 1). During the run‐in period, participants were not allowed to consume foods containing probiotics. After entering the intervention period, each subject was instructed to consume a sachet of probiotic (containing 0.1 g A6 of 5 × 10^10^ CFU and 3.4 g maltodextrin powder) or placebo (3.5 g maltodextrin powder without probiotic) once daily after either lunch or dinner for 8 weeks. Participants were advised to maintain their regular diet and exercise and they were not allowed to take any other probiotic or probiotic‐containing dietary supplements. Anti‐AR medications were prohibited in this study. Any adverse events or discomfort experienced by participants were noted down in their diaries. Questionnaires were collected using an online tool. Blood samples were collected before and after the intervention.

### 2.6 Outcomes

The primary outcome was TNSS. The secondary outcomes were rhinitis quality of life questionnaire (RQLQ) global score, eosinophil count, serum total IgE, sIgEs, and cytokines.

### 2.7 Questionnaires

TNSS was expressed as the sum of the scores for the four symptoms (nasal congestion, rhinorrhea, nasal itching, and sneezing). Each symptom was assessed using a 4‐point scale ranging from 0 (none) to 1 (mild), 2 (moderate), and 3 (severe). The total score was 12 points.^20^ The RQLQ is a validated scale used to evaluate the QoL. It consists of 28 questions that cover 7 domains, including activities, sleep, general practical problems, nasal issues, eye problems, and emotional state. All items are averaged to produce an overall score ranging from 0 (not at all) to 6 (extremely).^32^

### 2.8 Blood sample collection and blood index measurement

Blood samples were collected via venipuncture before the participants had breakfast in The First Medical Center of Chinese PLA General Hospital. Whole blood samples were sent immediately for blood cell count testing. Serum was then extracted and preserved in a −80‐degree refrigerator, waiting for IgE and cytokine measurements.

Blood cell count was assessed using a Sysmex xs‐800i hematology analyzer (Sysmex, Japan). Total IgE and sIgE levels were measured by ImmunoCAP 250 (Phadia, Sweden). IL‐4, IL‐5, IL‐9, IL‐10, IL‐13, INF‐γ, and TGF‐*β* were measured using ELISA kits (Shanghai Yuanju Biotechnology, China).

### 2.9 Statistical analysis

The Intent‐to‐treatment set (ITT) was used for analysis. The last observation carried forward (LOCF) was applied to fill the missing data. Continuous variables with normal or approximately normal distribution are described by mean (standard deviation), and continuous variables with skewed distribution are described as medians and interquartile ranges. Binary outcomes were reported as frequency (%). Paired sample *t*‐tests and paired sample rank sum tests were used for intra‐group comparisons. Independent *t*‐tests and Mann‐Whitney tests were used for inter‐group comparisons. The chi‐square test was used for categorical variables. Cohen's *d* was calculated to quantify the magnitude of differences, and interpreted as small if 0.2–0.5, moderate if 0.5–0.8, and large if > 0.8.^33^ *p* < 0.05 was considered as statistically significant. All statistical analyses were performed using SPSS 26.0.

## 3 RESULTS

### 3.1 Baseline characteristics

**FIGURE 2:**
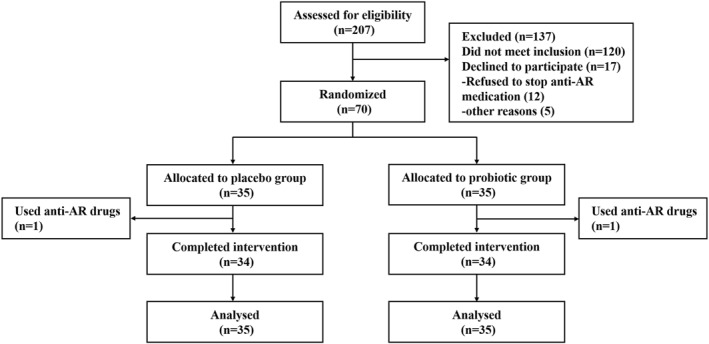
Participant flow diagram.

207 individuals were screened for eligibility, 137 of them did not meet the inclusion criteria, 12 patients refused to stop medication, and 5 patients quit for personal reasons. 70 patients with perennial AR were included (recruited 10 more patients than planned) and randomly assigned to take placebo or probiotics. Two patients dropped out because of taking medicine (Figure 2). At baseline, there were no statistically significant differences (*p* > 0.05) in gender, age, BMI, positivity of each sIgE, and TNSS between the groups (Table 1).

**TABLE 1:** Baseline characteristics.

	Placebo group (*n* = 35)	Probiotic group (*n* = 35)	*p*_0_
Gender (male: female)	13: 22	13: 22	1.000
Age (years)	27.2 (10.2)^a^	27.5 (9.2)	0.902
BMI (kg•m^−^^2^)	23.1 (3.4)^a^	23.6 (3.4)	0.546
Positivity of sIgE			
*Dp* [*n* (%)]	28 (80.0)	24 (68.6)	0.412
*Df* [*n* (%)]	27 (77.1)	24 (68.6)	0.730
*Cat* [*n* (%)]	11 (31.4)	12 (34.3)	1.000
*Dog* [*n* (%)]	6 (17.1)	7 (20.0)	1.000
TNSS	5.54 (2.93)^a^	5.97 (2.93)	0.543

Abbreviations: BMI, body mass index; *Df*, *Dermatophagoides farine*; *Dp*, *Dermatophagoides pteronyssinus*; sIgE, allergen‐specific immunoglobulin E; TNSS, total nasal symptom scores.

^a^ Data are expressed as mean (SD). *p*_0_ indicates the difference at baseline between the groups.

### 3.2 Effects of A6 on AR symptoms assessed by TNSS and RQLQ scales

As shown in Table 2, at week 4, TNSS exhibited a significant decrease compared to baseline in the probiotic group (*p* < 0.001) but not in the placebo group. Furthermore, the reduction in TNSS within the probiotic group was significantly greater than that of the placebo group [−2.26 (2.84) vs. −0.51 (3.05), *p* = 0.016, Cohen's *d* = 0.59], indicating a moderate effect size. At week 8, the reduction in TNSS was still more pronounced in the probiotic group compared with the placebo group [−3.11 (3.53) vs. −1.29 (3.34), *p* = 0.029, Cohen's *d* = 0.68], indicating a moderate effect size. Among individual symptoms, Nasal congestion and Rhinorrhea scores mirrored those of TNSS with greater reductions observed in the probiotic group following 4 and 8 weeks of intervention; meanwhile, no noteworthy differences were noted between groups for Itching and Sneezing scores.

**TABLE 2:** Effects of A6 on symptoms assessed by TNSS and RQLQ scales.

Parameter	Group	0 w	4 w	Change in 4 w	Cohen's *d*	8 w	Change in 8 w	Cohen's *d*
TNSS	Probiotic	5.97 (2.93)	3.71 (2.37)***	−2.26 (2.84)		2.88 (2.58)***	−3.11 (3.53)	
	Placebo	5.54 (2.93)	5.03 (3.15)	−0.51 (3.05)		4.26 (3.42)*	−1.29 (3.34)	
		*p*_0_ = 0.543		*p*_†_ = 0.016	0.59		*p*_†_ = 0.029	0.68
Nasal congestion	Probiotic	1.54 (0.74)	1.06 (0.73)***	−0.49 (0.74)		0.91 (0.82)***	−0.63 (0.94)	
	Placebo	1.29 (0.75)	1.46 (1.01)	0.17 (0.98)		1.11 (0.87)	−0.17 (0.75)	
		*p*_0_ = 0.154		*p*_†_ = 0.002	0.76		*p*_†_ = 0.028	0.54
Rhinorrhea	Probiotic	1.60 (0.91)	0.83 (0.71)***	−0.77 (1.03)		0.66 (0.76)***	−0.94 (1.11)	
	Placebo	1.26 (0.98)	1.14 (0.85)	−0.11 (1.21)		0.94 (1.00)	−0.31 (1.18)	
		*p*_0_ = 0.135		*p*_†_ = 0.017	0.59		*p*_†_ = 0.025	0.55
Itching	Probiotic	1.29 (0.93)	0.86 (0.85)**	−0.43 (0.95)		0.57 (0.70)***	−0.71 (1.13)	
	Placebo	1.46 (1.01)	1.20 (0.90)	−0.26 (0.95)		1.06 (1.00)*	−0.40 (0.98)	
		*p*_0_ = 0.462		*p*_†_ = 0.453	0.18		*p*_†_ = 0.217	0.29
Sneezing	Probiotic	1.54 (0.95)	0.97 (0.75)***	−0.57 (0.92)		0.71 (0.75)***	−0.83 (1.04)	
	Placebo	1.49 (0.92)	1.23 (0.97)	−0.26 (0.95)		1.14 (1.06)	−0.34 (1.14)	
		*p*_0_ = 0.799		*p*_†_ = 0.164	0.33		*p*_†_ = 0.067	0.45
	Probiotic	2.26 (1.21)	1.48 (0.96)***	−0.79 (0.99)		1.29 (0.80)***	−0.98 (1.20)	
RQLQ	Placebo	2.32 (1.20)	2.26 (1.32)	−0.06 (1.09)		1.68 (1.01)***	−0.64 (0.93)	
		*p*_0_ = 0.854		*p*_†_ = 0.005	0.70		*p*_†_ = 0.190	0.25

*Note*: Data are expressed as mean (SD). *p*_0_ denotes the difference at baseline between groups. *p*_†_ denotes the difference in change between groups. Cohen's *d* was calculated to quantify the magnitude of differences (changes in 4 and 8 w). * denotes the after‐intervention difference (4 w or 8 w) compared with baseline.

Abbreviations: RQLQ, rhinitis quality of life questionnaire; TNSS, total nasal symptom scores; w, week.

*p < 0.05, **p < 0.01, ***p < 0.001.

The overall trend of the RQLQ global score was consistent with TNSS. The decrease in the probiotic group was significantly greater than that in the placebo group [−0.79 (0.99) vs. −0.06 (1.09), *p* = 0.005, Cohen's *d* = 0.70] in week 4, indicating a moderate effect size; however, at week 8, no significant inter‐group difference was observed as both groups exhibited a notable reduction in RQLQ global score compared to baseline.

### 3.3 Effects of A6 on serum total IgE and sIgEs

Results of serum total IgE and sIgE levels are summarized in Table 3. After an 8‐week intervention, the decrease in serum total IgE was significantly greater in the probiotic group compared with the placebo group (−54.30 [105.18] vs. −10.52 [63.21] kU/L, *p* = 0.0498, Cohen's *d* = 0.50), indicating a moderate effect size. sIgE was analyzed if concentration ≥0.35 kU/L at baseline. No significant changes in serum sIgEs (*Dp*, *Df*, *cat*, and *dog*) were observed in either group compared with pre‐intervention levels. Additionally, no significant inter‐group difference was detected for any sIgE.

**TABLE 3:** Effects of A6 on serum total IgE and sIgE levels.

Parameter	Group	0 w	8 w	Change in 8 w	Cohen's *d*
Total IgE (kU/L)	Probiotic	153.00 (90.63, 494.25)	136.00 (80.75, 406.50)**	−54.30 (105.18)	
	Placebo	150.0 (87.90, 243.00)	142.0 (65.20, 242.00)	−10.52 (63.21)	
		*p*_0_ = 0.545		*p*_†_ = 0.0498	0.50
*Dp*‐sIgE (kU/L)	Probiotic	3.36 (1.67, 13.70)	4.11 (1.46, 15.10)	−0.79 (10.72)	
	Placebo	4.80 (2.29, 16.90)	3.62 (1.74, 15.30)	−1.23 (7.75)	
		*p*_0_ = 0.599		*p*_†_ = 0.864	0.05
*Df*‐sIgE (kU/L)	Probiotic	5.80 (2.78, 15.55)	6.43 (2.89, 17.20)	−1.40 (6.79)	
	Placebo	10.92 (2.85, 28.70)	12.20 (2.62, 33.35)	−0.99 (9.11)	
		*p*_0_ = 0.466		*p*_†_ = 0.859	0.06
*Cat*‐sIgE (kU/L)	Probiotic	1.44 (0.45, 25.70)	1.35 (0.67, 27.75)	2.13 (7.69)	
	Placebo	0.92 (0.56, 4.69)	0.79 (0.30, 8.28)	0.22 (2.96)	
		*p*_0_ = 0.622		*p*_†_ = 0.448	0.32
*Dog*‐sIgE (kU/L)	Probiotic	1.08 (0.42, 7.27)	1.73 (0.44, 9.00)	−0.95 (1.32)	
	Placebo	1.09 (0.82, 2.16)	1.32 (0.70, 4.53)	0.95 (2.77)	
		*p*_0_ = 0.935		*p*_†_ = 0.995	0.46

*Note*: Data are expressed as mean (SD) or median (25th percentile, 75th percentile). *p*_0_ denotes the difference at baseline between groups. *p*_†_ denotes the difference in change between groups. Cohen's *d* was calculated to quantify the magnitude of differences (changes in 8 w). * denotes the after‐intervention difference (8 w) compared with baseline.

Abbreviations: *Df*, *Dermatophagoides farine*; *Dp*, *Dermatophagoides pteronyssinus*; IgE, immunoglobulin E; sIgE, allergen‐specific IgE; w, week.

*p < 0.05, **p < 0.01, ***p < 0.001.

### 3.4 Effects of A6 on blood eosinophil count and serum cytokines

Table 4 shows the results of Blood eosinophil count, Th1, Th2, and Treg cell signature cytokines. After an 8‐week intervention, there were no statistically significant differences in blood eosinophil count and most cytokines (including IL‐4, IL‐5, IL‐9, IL‐10, IFN‐γ, and TGF‐*β*) in the A6 group compared with baseline, as well as in the placebo group. Only IL‐13 decreased significantly in the probiotic group (*p* < 0.01), and the decrease in serum IL‐13 was significantly greater than that in the placebo group (2.81 [14.29] vs. −8.07 [14.50] pg/mL, *p* = 0.004, Cohen's *d* = 0.76), indicating a moderate effect size.

**TABLE 4:** Effects of A6 on blood eosinophil count and serum cytokine levels.

Parameter	Placebo				Probiotic		*p*_0_	*p*_†_	Cohen's *d*
	0 w	8 w	Change in 8 w	0 w	8 w	Change in 8 w			
Blood eosinophil count (/μL)	154.52 (110.54)	165.49 (140.14)	10.97 (134.69)	186.06 (150.29)	168.79 (121.16)	−17.27 (157.03)	0.345	0.444	0.19
Th2 cytokines									
IL‐4 (pg/mL)	45.31 (18.46)	44.47 (16.75)	−0.84 (22.13)	42.60 (17.75)	44.00 (15.86)	1.40 (18.22)	0.551	0.660	0.11
IL‐5 (pg/mL)	70.82 (28.13)	63.50 (25.11)	−7.32 (39.68)	68.38 (26.53)	72.65 (25.46)	4.27 (37.48)	0.722	0.235	0.33
IL‐9 (pg/mL)	48.20 (13.40)	44.96 (16.17)	−3.24 (21.29)	45.64 (14.52)	47.05 (14.89)	1.41 (17.36)	0.467	0.341	0.24
IL‐13 (pg/mL)	30.39 (9.84)	33.20 (12.52)	2.81 (14.19)	35.65 (12.79)	27.58 (8.09)**	−8.07 (14.50)	0.069	0.004	0.76
Th1 cytokine									
INF‐*γ* (pg/mL)	695.54 (176.91)	766.79 (243.47)	70.85 (223.15)	685.78 (227.85)	703.98 (221.02)	18.20 (302.07)	0.843	0.433	0.20
Treg cytokines									
IL‐10 (pg/mL)	135.08 (142.68)	142.75 (151.48)	7.67 (67.52)	152.08 (154.19)	136.14 (131.05)	−15.94 (87.56)	0.649	0.234	0.30
TGF‐*β* (pg/mL)	3286.33 (1251.18)	3038.00 (1125.89)	−248.33 (1362.50)	2696.22 (881.65)	2882.24 (953.62)	186.02 (1308.31)	0.035	0.198	0.36

*Note*: Data are expressed as mean (SD). *p*_0_ denotes the difference at baseline between groups. *p*_†_ denotes the difference in change between groups. Cohen's *d* was calculated to quantify the magnitude of differences (changes in 8 w). * denotes the after‐intervention difference (8 w) compared with baseline.

Abbreviations: IL, interleukin; INF, interferon; TGF, transforming growth factor; Th, T helper; w, week.

*p < 0.05, **p < 0.01, ***p < 0.001.

No clinically significant adverse events were reported throughout the study period.

## 4 DISCUSSION

This randomized, double‐blind, placebo‐controlled trial showed that an 8‐week intervention of *Bifidobacterium animalis* subsp*. lactis* A6 (A6) significantly ameliorated symptoms of perennial AR, as evidenced by the decrease of TNSS and RQLQ global score, especially for Nasal congestion and Rhinorrhea. Furthermore, serum total IgE and IL‐13 levels also significantly decreased. No adverse events were reported during the study period. Our results suggest that A6 has the potential to alleviate perennial AR by inhibiting serum total IgE and IL‐13 levels.

TNSS is often considered an appropriate primary efficacy endpoint for AR^34^, which has also been widely used in previous RCTs.^20^, ^35^, ^36^ Furthermore, AR also affects QoL, with RQLQ being a validated scale^37^ widely used in previous RCTs.^32^, ^36^, ^38^ Therefore, TNSS was selected to be the primary outcome in our study, and RQLQ was used to assess the QoL. Several RCTs have discovered the remarkable efficacy of probiotics on AR symptoms^24^, ^39^ and QoL.^24^, ^40^ However, others have found no beneficial effect.^41^, ^42^ A recent meta‐analysis showed that probiotics significantly relieved AR symptoms (standardized mean difference [SMD], −0.29, 95% confidence interval (CI) [−0.44, −0.13]; *p* = 0.0003, *I* ^2^ = 89%), decreased RQLQ scores compared with the control group (SMD, −0.64, 95% CI [−0.79, −0.49], *p* < 0.00001, *I* ^2^ = 97%).^16^ The efficacy of A6 is generally comparable with that of the recent meta‐analysis (Table 2). However, the results can be affected by non‐allergen factors such as season, circadian rhythms,^43^ and medication choice.^8^ Monotherapy of probiotics significantly relieved AR symptoms (SMD, −0.73, 95% CI [−1.05, −0.42], *p* < 0.00001, *I* ^2^ = 93%); conversely, treatments combined with medication showed differing effectiveness (SMD, −0.15, 95% CI [−0.32, −0.03], *p* = 0.10, *I* ^2^ = 61%), as indicated by the subgroup analysis in the meta‐analysis.^16^ Thus, the “without anti‐AR medications” design of our study can independently evaluate the efficacy of A6. Additionally, our trial was implemented during winter in Beijing, largely avoiding the effects of seasonal inhaled allergens, although not all seasonal variations could be avoided.

IgE is an important mediator in AR pathology.^44^ In existing RCTs, no probiotic administrations demonstrated a significant reduction in serum total IgE than placebo.^14^, ^15^, ^16^, ^45^ To our knowledge, A6 is the first probiotic proven to have a statistically significant effect on serum total IgE in AR. However, A6 did not significantly alter serum sIgE levels. A previous study showed that a mixture of *Bifidobacterium longum* and *Lactobacillus plantarum* significantly reduced serum *Df*‐sIgE in adults with perennial AR.^20^ However, meta‐analysis showed no significant group difference (SMD, 0.09, 95% CI [−0.16, 0.34], *I* ^2^ = 0%).^16^ Thus, the results of sIgEs in our study were generally consistent with the majority of previous studies. The levels of sIgE antibodies are not only influenced by the immune system but also by external factors such as the frequency of allergen contact or lifestyle.^46^ A previous study showed that the placebo group with perennial AR had significant changes in serum anti‐house dust mite levels over time.^47^ Hence, complex confounders may weaken the effects of A6 on serum sIgE levels. In past studies on perennial AR, probiotics didn't significantly alter blood eosinophils compared with placebos,^20^, ^39^, ^42^, ^47^, ^48^, ^49^ which was also observed in this study.

Dysregulation of the Th1/Th2 balance may result in excessive activation of Th2 cells, leading to the induction of AR.^50^ Treg signature cytokines also play an important role in regulating Th1/Th2 balance.^7^ Evaluating this complex immune network requires systematically considering multiple cytokines. Part of the previous studies have found that probiotics can significantly improve some signature cytokines. For instance, a mixture of *Bifidobacterium longum* and *Lactobacillus plantarum* alleviated AR by inducing IL‐10 expression. *Bifidobacterium lactis* NCC2818 significantly reduced serum IL‐5 and IL‐13 levels in seasonal AR adults.^23^ Our results suggested that only serum IL‐13 level decreased significantly. As a member of the Th2 cell signature cytokines,^7^ IL‐13 plays a crucial role in airway bronchial hyper‐responsiveness^51^ and late‐phase responses during AR onset.^52^ The specificity of strains may be the reason why various probiotics play different regulatory roles.^53^ Duration of intervention and dose of probiotics may also influence the results. However, A6 significantly reduced serum concentrations of IL‐13, suggesting that it may have the same regulatory mechanism as *Bifidobacterium lactis* NCC2818.

This study has several strengths. First, a “without anti‐AR medications” and “winter‐intervention” design reduced confounding factors. Second, we conducted a relatively comprehensive assessment of changes in multiple Th cell signature cytokines to evaluate A6's impact on immune balance.

Nevertheless, our study had limitations. First, given that the subjects recruited were allergic to the four most common perennial inhalation allergens in Beijing, the results may not be generalizable to all perennial AR populations. More meticulously designed tests for allergens, encompassing other perennial allergens and seasonal allergens, are required in future studies to enhance the reliability and generalizability of the findings. Second, there was a lack of a follow‐up period to assess persistent changes in AR patients after discontinuation of A6 administration. Third, due to the complex complications of AR,^2^ TNSS and RQLQ global score alone cannot establish the most systematic evaluation system for subjective indexes. Various AR symptom assessment tools, such as the criteria recommended by Allergic Rhinitis and its Impact on Asthma (ARIA) 2008 for classifying the severity of rhinitis symptoms (from mild to severe)^1^ should also be considered. Fourth, given that the secondary outcomes in this study are exploratory in nature and lack multiple comparisons, these findings should be interpreted as preliminary. Finally, although A6 alone significantly alleviated AR in our study, we did not compare its efficacy with primary clinical treatments; its clinical value remains unclear.

## 5 CONCLUSION

In conclusion, this study demonstrated that 8 weeks of probiotic A6 administration statistically significantly alleviated rhinitis symptoms and improved QoL in adults with perennial AR. Reduction in serum total IgE and IL‐13 might be associated with A6 treatment. All the significantly changed indexes indicated a moderate effect size, suggesting that A6 may elicit clinically meaningful benefits for AR to a certain degree. However, these findings require validation through further confirmatory studies.

## AUTHOR CONTRIBUTIONS

**Langrun Wang**: Conceptualization; methodology; software; data curation; formal analysis; writing—original draft; visualization; writing—review and editing; project administration; investigation. **Shiwen Zhou**: Data curation; validation; investigation; project administration; formal analysis. **Huiyu Chen**: Data curation; writing—original draft; writing—review and editing; validation. **Chao Zhang**: Software; project administration; investigation. **Meiwen Sun**: Data curation; investigation; project administration. **Qi Zhang**: Data curation; investigation; project administration. **Yinghua Liu**: Resources; project administration. **Shaoqi Shi**: Investigation; project administration. **Shaoyang Ge**: Project administration; resources. **Juan Chen**: Writing—review and editing; investigation; validation. **Yanling Hao**: Writing—review and editing; validation. **Yong Zhang**: Resources; project administration. **Bing Fang**: Data curation; visualization. **Jingjing He**: Conceptualization; methodology; software; formal analysis; writing—original draft; supervision; writing—review and editing; investigation; project administration; validation; visualization. **Ran Wang**: Methodology; resources; supervision; writing—review and editing; investigation; project administration; funding acquisition.

## CONFLICT OF INTEREST STATEMENT

The authors declare no conflicts of interest.

## Data Availability

The datasets generated for this study are available on request to the corresponding author.
